# Inflammatory Myofibroblastic Tumor of the Central Airways: Case Report and Review of Diagnostic and Therapeutic Strategies

**DOI:** 10.1155/crpu/3123893

**Published:** 2026-09-02

**Authors:** Ahmed Ehab, Naoufal Benabdallah, Ibrahim Yasin, Ulrich Klein, Erik Büscher, Arndt Borkhardt, Kaid Darwiche

**Affiliations:** ^1^ Pulmonary Medicine Department, DGD Hemer Lung Center, Hemer, Germany; ^2^ Pulmonary Medicine Department, Mansoura University, Mansoura, Egypt, mans.edu.eg; ^3^ Thoracic Surgery Department, DGD Hemer Lung Center, Hemer, Germany; ^4^ Department of Pulmonary Medicine, University Medicine Essen-Ruhrlandklinik, Essen, Germany; ^5^ Department of Pediatric Oncology, Hematology and Clinical Immunology, Medical Faculty, Heinrich Heine University, Duesseldorf, Germany, uni-duesseldorf.de

**Keywords:** airway obstruction, bronchoscopy, inflammatory myofibroblastic tumor, interventional pneumology, trachea

## Abstract

Inflammatory myofibroblastic tumors (IMTs) are rare mesenchymal neoplasms with intermediate malignant potential. While most commonly arising in the lung, airway involvement is exceedingly uncommon. Endobronchial IMTs typically manifest with symptoms of airway obstruction, including dyspnea, wheezing, and stridor. Diagnosis involves radiological imaging, bronchoscopic evaluation, and histopathological analysis. Additionally, assessment of anaplastic lymphoma kinase (ALK) status is particularly important in young and pediatric patients, as ALK‐targeted therapies constitute a viable treatment option for tumors harboring ALK rearrangements or other actionable kinase fusions. Complete surgical resection remains the gold standard for treatment; however, bronchoscopic resection may be an effective option in selected cases. Herein, we describe two cases of IMTs involving the airways—one in an adult and one in a child—both presenting with significant airway obstruction and managed with bronchoscopic resection.

## 1. Introduction

Primary tracheal neoplasms are extremely uncommon, accounting for less than 0.2% of all adult respiratory tract tumors, with benign tracheal tumors being more common in children. Among malignant lesions, squamous cell carcinoma and adenoid cystic carcinoma are the most prevalent histological subtypes [1].

Inflammatory myofibroblastic tumors (IMTs) are rare entities, most commonly arising within the lung parenchyma. Airway involvement accounts for only 0.04%–0.07% of all respiratory tract tumors [2].

Herein, we present a case series of two patients with tracheal tumors; both were histologically confirmed as IMTs.

## 2. Case Report/Case Presentation

### 2.1. Case 1

**Figure 1:**
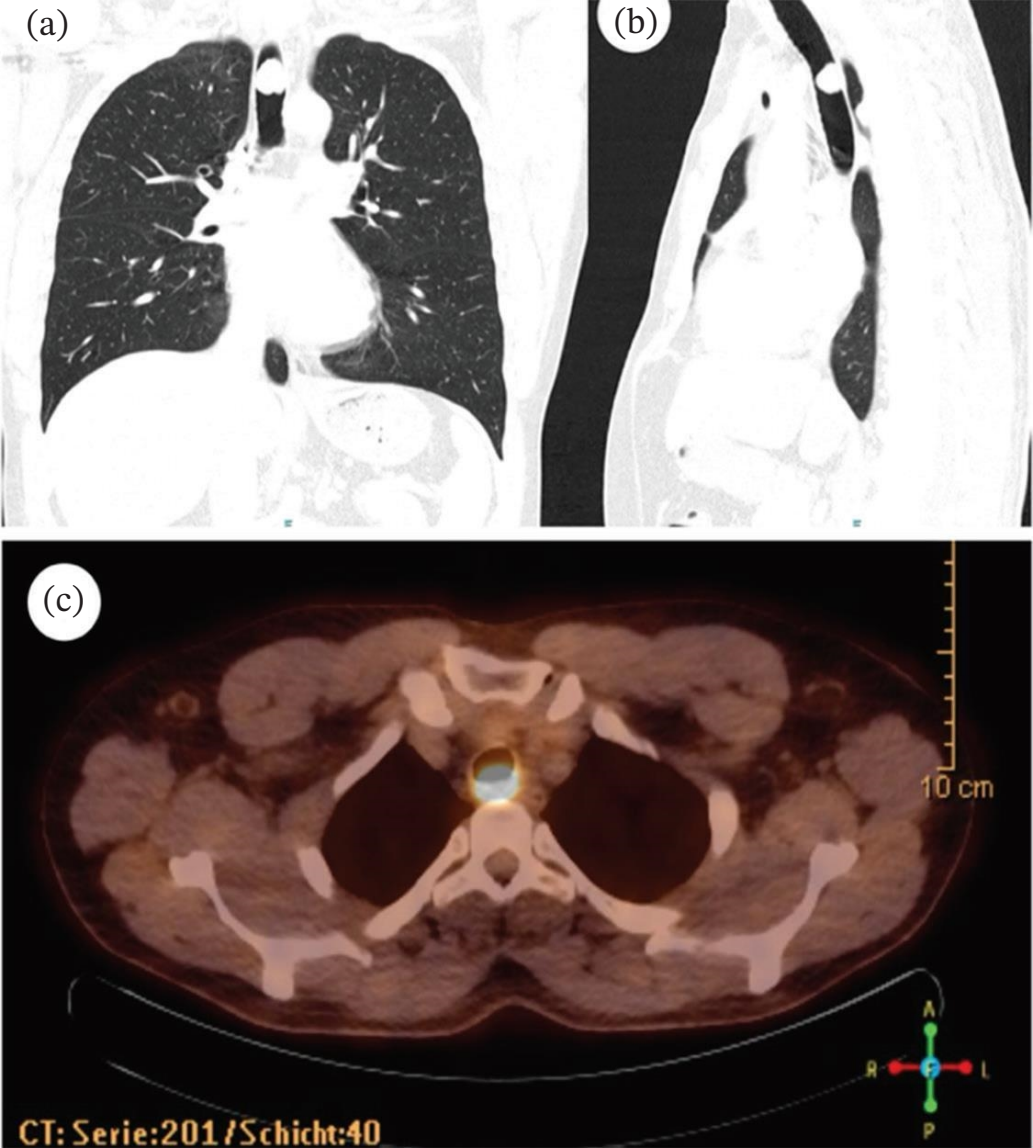
(a, b) CT chest (coronal and sagittal planes): broad‐based tumor arising from the pars membranacea of the trachea with near‐total obstruction of the lumen. (c) PET‐CT: PET‐CT positive polypoid intraluminal tracheal lesion.

A 42‐year‐old man with no relevant medical history presented with progressive exertional dyspnea. He denied cough, hemoptysis, or weight loss. Physical examination was unremarkable except for an inspiratory stridor. Computed tomography (CT) of the chest showed a large intraluminal tracheal mass causing near‐complete airway obstruction (shown in Figure 1a,b).

**Figure 2:**
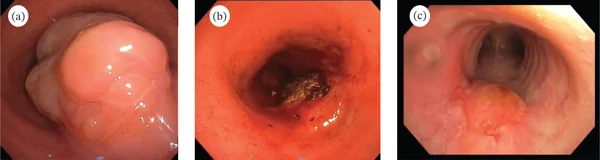
(a, b) Endoscopic appearance before and after excision and APC hemostasis. (c) 8‐week bronchoscopic follow‐up revealed no evidence of local tumor progression.

**Figure 3:**
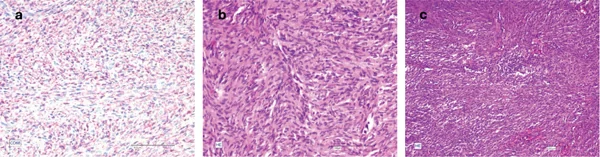
Histopathological findings of an inflammatory myofibroblastic tumor (IMT). (a) Immunohistochemistry (100 *μ*m): diffuse positivity in lesional spindle cells and stromal macrophages. (b) H&E (100 *μ*m): bland spindle cell proliferation in intersecting fascicles within a myxoid‐to‐collagenous stroma. (c) H&E (200 *μ*m): zonal architecture with spindle cell fascicles and mixed inflammatory infiltrate of lymphocytes, plasma cells, and lymphoid aggregates.

Bronchoscopic examination under general anesthesia revealed a broad‐based tumor originating from the membranous portion of the trachea, resulting in near‐complete occlusion of the airway lumen. The lesion was located approximately 4 cm above the main carina, while the endobronchial tree appeared normal. There was no evidence of esophageal wall involvement as assessed by bronchoscopic inspection of the esophagus. Local bronchoscopic debulking was performed using a snare and rigid forceps, followed by local application of argon plasma coagulation (APC) for coagulation of the tumor base (Figure 2b). Rapid on‐site cytology (ROSE) revealed the presence of spindle‐shaped cells. Subsequent histopathological and immunohistochemical analyses confirmed the diagnosis of an IMT with negative anaplastic lymphoma kinase (ALK) staining (Figure 3). The case was reviewed at a multidisciplinary tumor board, where definitive surgical resection was recommended. Positron emission tomography–computed tomography (PET‐CT) revealed a PET‐avid, polypoid intraluminal tracheal lesion dorsally located at the level of the T2–T3 vertebrae, with no evidence of additional abnormalities (shown in Figure 1c).

Following the bronchoscopic intervention, the patient showed marked symptomatic improvement. However, he declined the surgical resection and preferred close surveillance with regular follow‐up instead. At 8‐week follow‐up, bronchoscopic evaluation demonstrated no evidence of local tumor progression. Nevertheless, a subsequent surveillance bronchoscopy revealed recurrence of the tracheal tumor, resulting in subtotal tracheal stenosis. Consequently, surgical resection was rediscussed with the patient, who subsequently consented to surgery. Partial resection of the middle trachea was successfully performed, including complete tumor excision and end‐to‐end tracheal anastomosis. Histopathological examination confirmed the diagnosis of IMT.

### 2.2. Case 2

A previously healthy 10‐year‐old boy presented with progressive dyspnea and cough of several weeks duration. Physical examination of the chest revealed reduced breath sound over the left hemithorax. Bronchoscpic evaluation demonstrated a broad‐based endobronchial lesion obstructing the left main bronchus. Histopathological analysis confirmed the IMT. Immunohistochemistry showed strong cytoplasmic ALK‐1 positivity with weak smooth muscle actin (SMA) expression and negative staining for desmin, CD99, S100, and pancytokeratin, consistent with IMT. Targeted therapy with crizotinib (200 mg/day) was initiated in combination with prednisolone and celecoxib. The patient exhibited prompt clinical improvement, allowing tapering and discontinuation of prednisolone within the first months, while crizotinib plus celecoxib was continued as maintenance therapy. No clinically relevant adverse effects attributable to crizotinib were observed in this patient during the treatment and follow‐up period.

**Figure 4:**
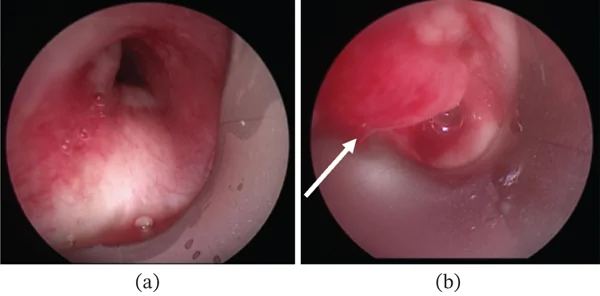
(a) Rigid bronchoscopy of the left main bronchus demonstrating marked endoluminal obstruction. (b) Near‐total occlusion of the left upper lobe orifice during tumor recurrence (white arrow).

Despite sustained clinical well‐being under treatment, surveillance bronchoscopy after 2 years revealed a local recurrence: a tumor focus along the medial wall of the left main bronchus causing approximately 50% luminal narrowing of the main bronchus and near‐complete occlusion (~95%) of the upper lobe bronchus orifice (shown in Figure 4).

Management options, including left pneumonectomy, were discussed in a multidisciplinary setting. Considering the morbidity of pneumonectomy in a child and the endoluminal localization of disease, the team, together with the patient and his parents, elected the local endoscopic management. Rigid bronchoscopy with mechanical debulking was performed, followed by three sessions of endobronchial cryotherapy over the subsequent months. After these procedures, airway patency was restored without significant residual stenosis, and repeated histology demonstrated no viable tumor and only chronic bronchitis.

Crizotinib and celecoxib were continued thereafter for a total treatment duration of approximately 36 months, after which all antineoplastic medication was discontinued. Serial surveillance with flexible bronchoscopy consistently revealed no evidence of tumor recurrence, showing only features of chronic bronchitis. Cross‐sectional imaging, including thoracic CT and a subsequent PET‐MRI performed 5 years later, demonstrated no signs of disease recurrence. The patient remained asymptomatic, physically active (regular skateboarding) and fully engaged in school, with unremarkable clinical examinations and no regular medication apart from treatment for seasonal allergic rhinitis. In view of the durable remission, follow‐up intervals were extended to annual visits.

## 3. Discussion

IMTs are a rare mesenchymal tumor with intermediate malignant potential. According to the World Health Organization (WHO), IMTs are characterized by proliferation of myofibroblastic spindle cells accompanied by infiltration with inflammatory cells, for example, plasma cells, lymphocytes, and eosinophils, hence the controversy in terminology from inflammatory pseudotumor [1], [3]. While initially thought to represent a reactive or inflammatory process, accumulating evidence—including recurrence, occasional metastasis, and clonal chromosomal abnormalities—now supports its neoplastic nature. ALK rearrangements are identified in nearly half of cases, with additional gene fusions involving ROS1, PDGFR*β*, and NTRK further reinforcing this concept [1], [4]. In this context, IMTs typically exhibit strong expression of vimentin and SMA, while desmin may be variably expressed. A distinctive finding—especially in younger patients—is the presence of ALK expression in approximately 50% of cases. The absence of immunoreactivity for CD34, S100, and CD117 plays a key role in excluding other spindle cell tumors from the differential diagnosis [1].

Reported cases range in age from 3 to 72 years, with more than 50% diagnosed in individuals younger than 40 years. Endobronchial IMTs are more commonly described in pediatric patients, as observed in the second case, whereas occurrence in adults is less frequent, as illustrated by the first case [5].

Symptoms of IMT vary depending on the anatomical site of involvement. Airway affection typically presents with signs of progressive airway obstruction, including dyspnea, persistent cough, wheezing, and stridor. These nonspecific symptoms often mimic common respiratory disorders such as asthma, which may result in delayed diagnosis [6].

CT of the chest typically demonstrated a nonenhancing, homogenous mass with infiltrative yet distinct borders. In airway involvement, IMTs often present as well‐circumscribed endoluminal soft‐tissue masses, which may cause partial or complete airway obstruction. Both calcification and necrosis are uncommon findings [1].

As demonstrated in the first case report, 18F‐fluorodeoxyglucose (FDG) PET/CT may be useful for identifying the primary tumor and assessing potential distant metastases. Tracheal IMTs typically exhibit increased metabolic activity on PET imaging, which may mimic malignant lesions. As FDG uptake reflects both neoplastic and inflammatory processes, PET/CT findings should always be interpreted in conjunction with histopathological evaluation [1], [7].

In case of IMT involvement of the airways, bronchoscopy is essential both for obtaining a definitive histopathological diagnosis and, in selected cases, for definitive therapeutic management. IMTs typically present as well‐circumscribed, polypoid, or lobulated endoluminal masses that may partially or completely obstruct the airway. The surface is often smooth, and vascularization may vary, sometimes posing a bleeding risk during biopsy [6], [1].

Variable treatment options were described in the literature; however, given their locally invasive behavior, reported recurrence rates of up to 40%, and the potential risk of metastasis, complete surgical resection with end‐to‐end anastomosis remains the treatment of choice for airway IMTs whenever feasible. Achieving negative resection margins offers the highest likelihood of cure [1], [8].

Radiotherapy may be used perioperatively—either neoadjuvantly to reduce tumor size or adjuvantly in cases of close or positive surgical margins. In nonoperable cases, it can be considered alone or with systemic therapy. However, due to the rarity of IMTs, the indications for radiotherapy are not yet well established, and current evidence is largely limited to case reports and small case series [9].

Endoscopic intervention demonstrated a minimally invasive therapeutic option with low procedural morbidity for tracheal and endobronchial IMTs, particularly when the lesion is confined to the airway lumen without evidence of transmural invasion. Techniques such as rigid bronchoscopy, laser resection, cryotherapy, or APC can be employed for tumor debulking or even complete resection, allowing for rapid restoration of airway patency and symptom relief [6, 10]. However, it may represent an alternative therapeutic option in carefully selected patients, particularly those with endoluminal airway‐confined lesions, patients who are medically unfit for surgery, or cases in which lung‐preserving strategies are being considered. Such approaches should be undertaken only after thorough multidisciplinary discussion and consensus agreement.

In a recent case series by Jeong et al., involving eight adult patients, rigid bronchoscopy combined with laser excision achieved macroscopic complete resection in five of eight cases, with no procedure‐related mortality and no recurrence during a median follow‐up of 18.8 months [11]. This approach is especially valuable in patients who are poor surgical candidates or require urgent airway stabilization.

As demonstrated in the first case, bronchoscopic intervention, including endobronchial recanalization, was mandatory to achieve immediate relief of acute obstructive symptoms. However, this was not intended as a definitive treatment option. In the second case, bronchoscopic debulking combined with strict endoscopic surveillance and scheduled cryotherapy, in addition to ALK inhibition for ALK‐positive endobronchial IMT, served as an effective lung‐preserving strategy. Nevertheless, bronchoscopic surveillance revealed local recurrence despite maintenance treatment with an ALK inhibitor, indicating that systemic targeted therapy alone may not be sufficient to prevent local recurrence. In this setting, repeated endoscopic interventions, including bronchoscopic debulking and cryotherapy, successfully achieved sustained local disease control. Regarding the long‐term outcomes following bronchoscopic intervention, a case series of three patients demonstrated encouraging results after bronchoscopic debulking. Two patients remained free of radiological and bronchoscopic recurrence after 38 and 40 months of follow‐up, respectively. In the third patient, local recurrence developed and was successfully managed with surgical resection. These findings suggest that, in carefully selected patients with purely endoluminal IMTs, bronchoscopic intervention may represent an effective lung‐sparing therapeutic approach, while surgery remains an important treatment option for recurrent or persistent disease [12].

Other treatment modalities include systemic steroids or chemotherapeutic agents such as cyclophosphamide, methotrexate, or cyclosporine, which have been employed in patients with distant metastases, however with variable clinical outcomes. Moreover, target biological therapies have been recently introduced particularly inhibitors of the kinase domain of ALK protein; for example, crizotinib—as described in the second case report—has demonstrated a potential positive results in the presence of ALK rearrangement or other actionable kinase fusion, resulting in favorable therapeutic responses. The overall response rate to crizotinib in 14 pediatric patients with inoperable or metastatic ALK‐positive IMT was 86%, including a complete response in 36% of patients and a partial response in 50% [13]. In another study conducted on nine patients, the objective response rate was 67%, with a median treatment duration of 3 years [14]. In the second case, celecoxib was added empirically as an anti‐inflammatory and potential antiangiogenic adjunct to crizotinib. This decision was informed by the known inflammatory biology of IMT and published evidence demonstrating coexpression of COX‐2 and vascular endothelial growth factor (VEGF) in IMT tissue. Applebaum et al. examined IMT specimens within a Children′s Oncology Group study and identified COX‐2 and VEGF expression across analyzed samples [15], suggesting that these mediators may contribute to tumor growth and providing a biological rationale for NSAID or COX‐2 inhibitor therapy in selected cases. Consistent with this, isolated case reports have described responses of recurrent or unresectable IMTs to COX‐2‐directed therapy, including cases achieving durable remission under celecoxib‐containing regimens, both in ALK‐positive disease treated [16] in combination with targeted therapy and in ALK‐negative IMT as monotherapy [17]. It must be acknowledged, however, that the evidence base for COX‐2 inhibition in IMT remains limited to mechanistic data and anecdotal case reports, and this approach is not a prospectively validated component of standard IMT therapy. Its use in our patient was therefore adjunctive and empirical, and its contribution to the observed clinical response cannot be definitively quantified independently of crizotinib. However, it should be emphasized that the available evidence supporting treatment with crizotinib is derived primarily from case reports, case series, and retrospective studies, with no randomized controlled trials currently available to establish its efficacy, the optimal duration of therapy, or its long‐term safety profile. This is understandable given the rarity of the tumor and the inherent challenges of conducting adequately powered controlled trials in such a rare disease. Additionally, although crizotinib is generally well tolerated in pediatric patients, most adverse effects are mild to moderate and can be managed with dose reduction or temporary treatment interruption. The most commonly reported toxicities include gastrointestinal symptoms (nausea, vomiting, and diarrhea), hepatotoxicity with elevated transaminases, visual disturbances, peripheral edema, fatigue, and hematologic abnormalities. Among these, neutropenia represents the most frequent Grade 3–4 adverse event and is the leading cause of treatment modification. Compared with adults, pediatric patients appear to experience higher rates of hematologic toxicity and transaminase elevation, highlighting the importance of regular monitoring of blood counts, liver function, and cardiac parameters during treatment [18].

Finally, this case series is limited by the small number of included patients (*n* = 2) and the lack of standardized posttreatment imaging protocols.

In conclusion, IMTs—although rare—should be considered in the differential diagnosis of endobronchial lesions in young adults. Due to their neoplastic nature, along with potential risk of recurrence and the rare possibility of metastatic spread, surgical resection remains the treatment of choice whenever tumor location, extent, and the patient′s clinical status allow safe intervention. In carefully selected cases of endobronchial IMTs without evidence of transmural invasion, who are unfit for definitive surgical resection, bronchoscopic management may represent an adequate minimally invasive alternative, providing rapid relief, final histological diagnosis, and favorable short‐ to mid‐term outcomes. However, regular surveillance bronchoscopy remains essential in this case. Additionally, targeted therapies have shown promising results in patients with ALK rearrangements with favorable therapeutic responses. However, important uncertainties remain regarding the optimal duration of treatment and long‐term safety.

## Author Contributions

Ahmed Ehab wrote the main manuscript text, prepared the figures, and reviewed the manuscript. Naoufal Benabdallah wrote the main manuscript text, prepared the figures, and reviewed the manuscript. Ibrahim Yasin reviewed the manuscript. Ulrich Klein reviewed the manuscript. Erik Büscher wrote the main manuscript text, prepared the figures, and reviewed the manuscript. Arndt Borkhardt reviewed the manuscript. Kaid Darwiche supervised and reviewed the manuscript.

## Funding

No funding was received for this manuscript.

## Ethics Statement

Written informed consent was obtained from both the adult patient and the parents of the minor patient for publication of this report and any accompanying images.

## Conflicts of Interest

The authors declare no conflicts of interest.

## Data Availability

The data that support the findings of this study are available on request from the corresponding author. The data are not publicly available due to privacy or ethical restrictions.
